# Anxiety, Reinforcement Sensitivity and Social Context in Accepting the Experience of Pain Among Rheumatoid Arthritis Patients

**DOI:** 10.3389/fpsyt.2020.554990

**Published:** 2020-11-25

**Authors:** Luis Pinel, Miguel A. Perez-Nieto, Marta Redondo, Luis Rodríguez-Rodríguez, Leticia León

**Affiliations:** ^1^Faculty of Education and Health, Camilo José Cela University, Madrid, Spain; ^2^Rheumatology Service, Hospital Clínico Universitario San Carlos, Madrid, Spain

**Keywords:** mindfulness, cognitive behavioral therapy, social influences, reinforcement sensitivity, anxiety, acceptance, chronic pain

## Abstract

**Background:** Acceptance has become one of the most widely studied processes regarding chronic pain because of its ability to influence participants' adaptation and coping responses. Leading researchers have found relationships between variables such as anxiety, reinforcement sensitivity, and the responses of the participants' environment to their behavior and acceptance. In contrast, few studies have been found that investigate the variables that predict the acceptance of pain. This study has set out to explore the relationships between pain-related anxiety, sensitivity to contingencies, and the punishment responses of significant people toward pain behaviors regarding pain acceptance.

**Methods:** With a view to fulfilling this purpose, a cohort of 62 participants with rheumatoid arthritis was chosen, and the subjects were assessed through the following self-report measures: Chronic Pain Acceptance Questionnaire, CPAQ; Pain Anxiety Symptoms Scale, PASS-20; The Sensitivity to Punishment and Sensitivity to Reward Questionnaire, SPSRQ, and The West Haven-Yale Multidimensional Pain Inventory, WHYMPI. The study's initial objectives were achieved by means of a stepwise multiple linear regression analysis.

**Results:** The linear regression analyses revealed a negative and significant correlation between anxiety, reinforcement sensitivity, and the significant persons' responses to pain behaviors and pain acceptance.

**Conclusion:** The results suggest that the identification of these variables might be important for addressing these participants' pain. Finally, the discussion focuses on our findings' implications as regards their use in clinical practice.

## Introduction

Chronic pain has latterly become an increasingly serious health issue, as reflected by the new steps taken by the World Health Organization, which has now classified this experience as a major illness (1). The foremost epidemiological studies indicate that chronic pain is highly prevalent worldwide (2–4). Its most serious consequences include impacting upon the quality of life of participants and their families, negative psychological effects, the disability it causes, problems associated with the loss of productivity at work, and the high socio-economic costs incurred by the health system (5–9). Therefore, and in view of these circumstances, it is understandable that chronic pain is now considered a major public health issue (10), becoming a key study topic for leading researchers in the field.

Acceptance has been one of the more widely studied pain-related variables in recent years, as it has rapidly become a significant process for the applied clinical context because of its ability to influence the manner in which participants adapt to and cope with the experience of responding to pain (11–17). On a theoretical level, we are dealing with a complex construct that scholars have interpreted differently (16). Nevertheless, the acceptance of pain has traditionally been defined as an individual's constant readiness to experience pain (thoughts, feelings, sensations, etc.) without offering any resistance, while channeling their behavior toward valuable goals and objectives (11). According to McCracken (16), this definition has two vital components for its understanding: openness or receptivity to pain, and involvement in activities. The component of openness means surrendering to sensorial aspects, to pain-related feelings, thoughts, and emotions. The second component of involvement refers to a subject's commitment to tailor their behavior according to their values and continue with their everyday activities despite the pain. Based on this conceptualization as our reference framework, we have found numerous studies that relate the acceptance of pain to more adaptive coping, being associated with a lower emotional, physical, and social function (11, 13, 18–22), a lower level of reported pain (23–25), less disability (26), and a reduced use of medication (12). Along these same lines, we find solid evidence to show how clinical interventions based on the acceptance of pain, such as acceptance and commitment therapy (ACT) (27) or mindfulness-based interventions (MBIs) (28), are more effective than processes already in place (29–34). The data available have shown that acceptance is a highly important variable in chronic pain, both at theoretical level and in the field of applied clinical treatment; nevertheless, we have found very few studies that have addressed the psychological variables that might predict higher levels of pain acceptance. Finding these predictors will help to improve the process of selecting the treatment to be followed with these participants.

Anxiety has been described in the literature as a significant factor in acceptance processes in different samples of participants with chronic pain. High levels of pain-related anxiety have therefore been associated with lower levels of openness toward the same, and less involvement in activities by the sufferer; in other words, there seems to be a strong, negative relationship with acceptance (11–13, 21, 35–37). Elsewhere, we encounter studies that address anxiety sensitivity (AS), which has been defined as a trait that predisposes someone to experience a fear of pain and develop anxiety disorders (38). Several scholars have posited that through its predisposition to the fear of pain, AS is directly related to the adoption of escape or avoidance behaviors (39–41). Experiential avoidance is a key pattern of behavior that is located at the other extreme from acceptance (42), whereby it may be argued that AS is indirectly related to the acceptance of this feeling. When we consider the findings of these studies as a whole, they all suggest that anxiety plays a crucial role as a predictor of low acceptance in contexts of chronic pain.

In addition, and in this same vein, there are two known neuropsychological systems that can impact upon avoidance and approach behaviors: the Behavioral Approach/Activation System (BAS) and the Behavioral Inhibition System (BIS). The most widely cited theory of the different approach-avoidance models is Reinforcement Sensitivity Theory (43, 44). This model has recently been reviewed within the field of chronic pain [to read the review, see (45)]. This model indicates that the guidelines for behavioral approach or avoidance in certain situations depend on contextual keys (internal or external), which predict the probability of receiving a reward or a punishment (46). The BAS is therefore triggered by the presence of keys that indicate the possibility of obtaining a reward, or of eliminating or reducing the likelihood of an aversive stimulus, while the BIS is triggered by the presence of keys that predict a punishment (e.g., pain, disability, catastrophic thoughts, and anxiety). Numerous researchers have found that participants with chronic pain record more BIS activity and less of BAS (47–51). These systems are in some way mutually inhibited, and their alternance can be explained by sensitivity in the presence of the aversive or appetitive stimulus (52). We have found certain studies that report that these participants are more sensitive to reinforcement than control groups (47, 53). An analysis of this information is expected to show that sensitivity to reinforcement and punishment is related to the adoption of behaviors of greater or lesser openness and involvement regarding pain, and therefore to its acceptance. Furthermore, sensitivity to punishment is also associated with less social activity and a lower probability of social support (54), with the latter being a highly important variable in coping with chronic pain (55, 56).

Related to this last point, research has focused its attention on interpersonal relationships involving participants with chronic pain, and more specifically within the family setting. According to the theory of operant conditioning, the immediate environment's response has the ability to promote behaviors of pain or well-being among participants with chronic pain (57). Many studies have reported that solicitous responses (e.g., expressions of support or concern, or instrumental support for the pain behavior) and punishing responses (e.g., expressions of frustration or irritation toward the pain behavior) by significant people close to the patient are linked to an increase in pain, lower levels of activity, more pain behaviors, more visits to the doctor, and greater disability (58–67). This means that significant people's reaction to these participants' pain behaviors may have an indirect impact on pain acceptance processes. Furthermore, relatively large studies involving participants with chronic pain have found a strong and negative relationship between solicitous and punishing responses and pain acceptance (68), maintaining its predictor value even a year after the medical intervention (69).

The information provided as theoretical underpinnings has informed this study designed to examine the relationship between pain-related anxiety, sensitivity toward punishment and reinforcement, significant people's response to pain behaviors, and its predictive capacity in terms of pain acceptance, due to the relationship shown by these variables in the aforementioned studies. Results will inform treatment decision-making and the standard of psychological care provided to people with chronic pain.

## Methods

### Participants

**Figure 1:**
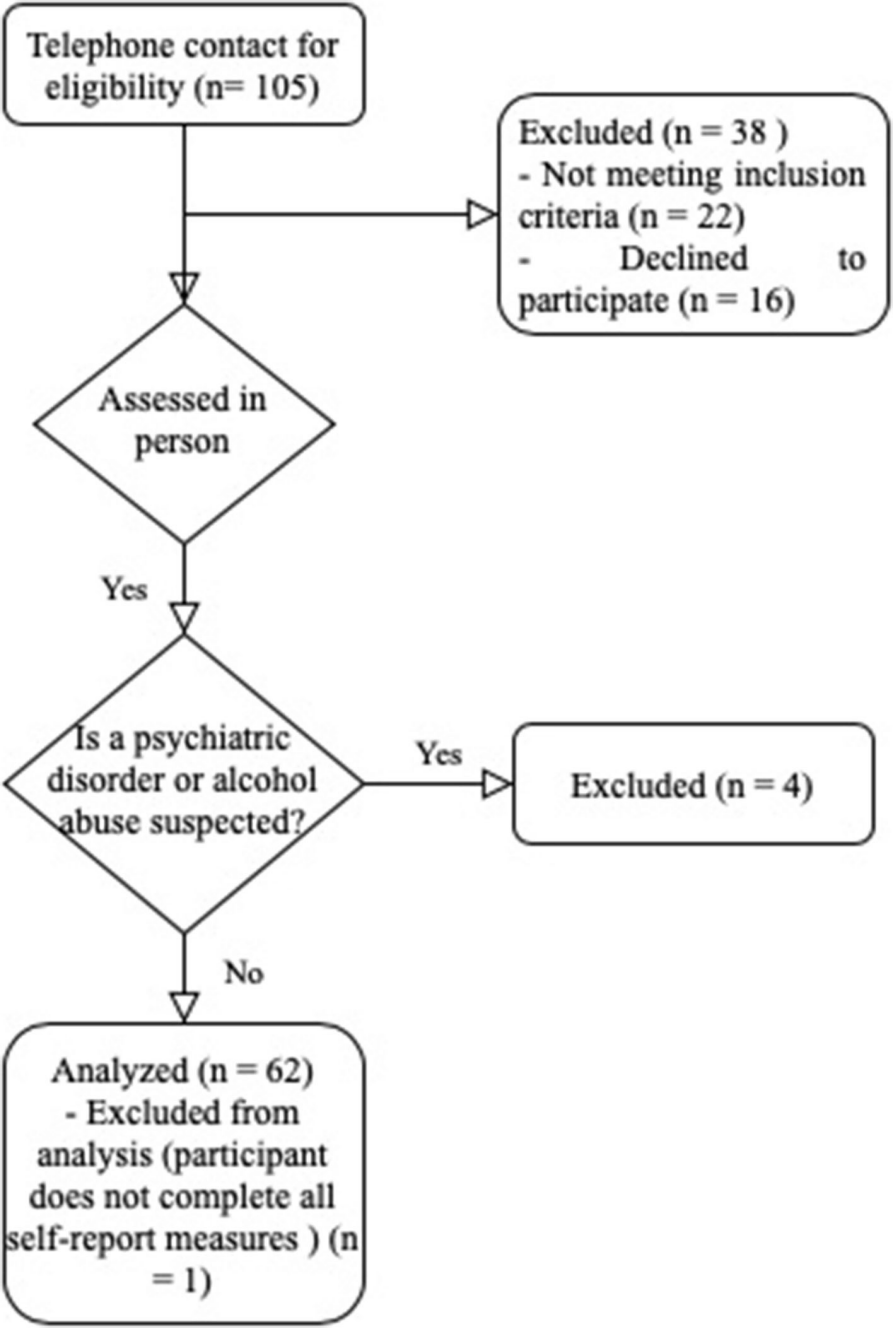
Study design flowchart.

The study was approved by the Research and Ethics Committee of CEIC Hospital Clínico San Carlos in Spain. Subjects eligible for the study were patients with rheumatoid arthritis (*n* = 62) participants who were undergoing treatment in the Department of Rheumatology at the hospital and at the Madrid Association of Participants with Rheumatoid Arthritis (AMAPAR, in its Spanish acronym). All the data required for the study were gathered between December 2015 and February 2017. Subjects were screened by phone about their interest of participation in the study, only participants with higher interest were selected for evaluation. Subjects who indicated that they were medically healthy, other than rheumatoid arthritis, aged ≥18 years willingness to give consent and participate in the study, were asked to meet the lead researcher on a face-to-face interview for individual assessment. The assessment was conducted individually in a single session by the same assessor, without any limit of time. On average, each session took one and a half hours. During the evaluation process participants were excluded if they had: (1) a history of psychiatric disorder such as major depressive disorder, obsessive-compulsive disorder or anxiety generalized disorder schizophrenia; (2) lack of motivation to complete the self-report measures; (3) or high levels of alcohol/substance abuse. Female patients who were pregnant or lactating women were not grounds for exclusion. The participants who refused to complete data on all self-report measures listed below were excluded from final sample (*n* = 6). Participant characteristics are presented in a table in results section (see Table 1). The study design is presented in Figure 1.

**Table 1:** Sociodemographic characteristics and clinical variables of the study participants.

**Characteristic**	**Frequency (*n*)**	**Percentage (%)**
**Sex**		
Female	13	21
Male	49	79
**Age in years** ***(M, SD)***	53.2 ± (11.2)	
**Marital status**		
Single	15	24.2
Married	31	50.0
Widowed	3	4.8
Divorced	7	11.3
Separated	6	9.7
**Education level**		
Primary	7	11.3
EGB or equivalent	7	11.3
Technical and vocational	10	16.1
Senior high school	17	27.4
University	15	24.2
Higher education	3	4.8
Unregulated studies	3	4.8
**Socioeconomic status**		
Low	13	21
Medium	43	69.3
High	6	9.7
**Time elapsed since the first medical diagnosis**		
Less than a year	4	6.4
Less than 3 years	4	6.5
Less than 5 years	2	3.2
Between 5 and 10 years	15	24.2
More than 10 years	37	59.7
**Pharmacological treatment**		
None	1	1.6
Biological agents (e.g., Infliximab, Abatacept, etc.).	2	3.2
FAMES (e.g., Metotrexato).	1	1.6
Corticosteroids	0	0
Anti-inflammatory drugs	1	1.6
Analgesic drugs	0	0
Others	2	3.2
Several of the above	55	88.7

*M, mean; SD, standard deviation*.

### Ethical Statement

The study protocol was approved by the Research and Ethics Committee of CEIC Hospital Clínico San Carlos in Spain, registration number 15.531-E. Date of communication: 10 December 2015. All participants signed a consent form after been informed about eligibility criteria and study procedures. The lead investigator monitored the data collection and informed consent process. Only participants who completed data for all self-report measures listed below were included in the sample and taken into consideration for the statistical analyses. Those who did not meet the conditions of eligibility previously mentioned were discarded.

### Procedure and Self-Report Measures

The self-report measures were administered on a face-to-face basis at the Department of Rheumatology at the Hospital Clínico San Carlos in Madrid (Spain) and at AMAPAR. Only five participants were assessed by videoconferencing via Skype. The participants were invited to take part in the study, and once they had voluntarily agreed to do so, they left their phone number for the initial contact. The lead researcher subsequently contacted the interested parties to give them information on the study and arrange a meeting. The assessment was conducted individually in a single session, without any limit of time, and always involved the same assessor. During the appointment, the participants signed the informed consent form and completed a socio-demographic questionnaire, answering questions about the nature of the pain, the time elapsed since their first diagnosis, and the medical treatment they were following. Finally, they also completed a series of self-report measures on the psychological variables to be studied, as described in what follows.

- **CPAQ**. *Chronic Pain Acceptance Questionnaire* [(16): Spanish version by Menéndez (70)]. It consists of 20 items that assess the acceptance of pain in participants with chronic pain. The questionnaire has two subscales: openness to pain and involvement in activities. The former refers to an individual's willingness to experience pain without putting up any resistance, while the latter assesses an individual's ability to take part in activities despite the pain. The answers involve a Likert-type scale from 0 (never true) to 6 (always true). Our sample recorded suitable levels of internal consistency reliability for the total scale (Cronbach's alpha = 0.690), according to the criteria proposed by Prieto (71).
- **PASS-20**. *Pain Anxiety Symptoms Scale* (72). This scale explores anxiety responses to pain: fear, escape/avoidance, physiological anxiety, and cognitive anxiety. It consists of 20 items with Likert-type answers ranging from 1 (never) to 5 (always). Our sample has recorded suitable criteria for internal consistency reliability through Cronbach's alpha coefficient (presented in brackets) for its five component subscales: fear (0.772), escape/avoidance (0.649), physiological anxiety (0.598), cognitive anxiety (0.811), and overall scale (0.880). They are suitable according to Prieto's criteria (71).
- **SPSRQ**. *The Sensitivity to Punishment and Sensitivity to Reward Questionnaire* (73). This is a self-report measure consisting of 48 items with a dichotomous (Yes/No) answer format. It is divided into two subscales, each with 24 items: sensitivity to reward (STR) (behaviors focusing on the search for reinforcers, such as the search for sensations, money, or power), and sensitivity to punishment (SP) (behaviors designed to avoid aversive stimuli or negative consequences, due to the possibility of harm or failure). Through Cronbach's alpha coefficient (presented in brackets), this study has recorded acceptable levels of reliability for STR (0.725) and good ones for SP (0.825), which were appropriate according to Prieto's criteria (71).
- **WHYMPI**. *The West Haven-Yale Multidimensional Pain Inventory* [(74); Spanish version by Ferrer (75)]. The study applied the second domain of the questionnaire corresponding to the subscale that assesses the reinforcing and punishing responses provided by the caregivers in response to a patient's pain behaviors. This section consists of 14 items with a Likert-type response format ranging from 0 (never) to 6 (very often). The measure has recorded good levels of reliability in our sample (Cronbach's alpha = 0.842) according to the criteria proposed by Prieto (71).

### Data Analysis

The data were coded and analyzed using version 25.0 of the SPSS statistical package. The goals considered here involved conducting multiple stepwise linear regression exploratory analyses. The predictor variables used were cognitive anxiety, reinforcement sensitivity, sensitivity to punishment, the reinforcements and punishments administered by the patient's carers in response to pain behaviors, as well as the variables to be controlled (age, time elapsed in months since the first symptoms of pain, socioeconomic status, and the current medical treatment being received). The acceptance of pain was used as the dependent variable or criterion variable. A series of prior tests were carried out to ensure compliance with the assumptions of normality, revealing a suitable distribution of the residuals. In terms of homoscedasticity, the Durbin-Watson results (1.656) are within the recommended range (2 ± 0.5). The tolerance values for the variables introduced were below 0.10, dismissing any problems of collinearity. These statistics therefore tell us that these data are suitable for a linear regression analysis.

## Results

### Sample Characteristics

The sample consisted of 62 participants (13M/49F), the mean age was 53.24 (SD = 11.29), ranging between 25 and 77. Half of the participants were married or in a long-term partnership (50%), followed by those that were single (24.2%), divorced (11.3%), separated (9.7%), and finally, widowed (4.8%). Regarding their educational status, many participants had completed primary (*n* = 7, 11, 3%), secondary school (*n* = 17, 27, 4%), or university (*n* = 18, 29%) studies. About a third of them had completed some type of tertiary or vocational education (*n* = 10, 16.1%). Altogether, 69.3% reported medium incomes (*n* = 43, 69.3%). The distribution of the time elapsed since the first medical diagnosis in our sample was less than a year (*n* = 4, 6.4%), <3 years (*n* = 4, 6.5%), <5 years (*n* = 2, 3.2%), between 5 and 10 years (*n* = 15, 24.2%), and more than 10 years (*n* = 37, 59.7%). Most of the participants were following a pharmacological treatment based on FAMES (*n* = 1, 1.6%), anti-inflammatory medication (*n* = 1, 1.6%), biological medication (*n* = 2, 3.2%), others (*n* = 2, 3.2%), and several of these (*n* = 55, 88.7%). Only 1.6% were not receiving any medical treatment. The characteristics of the study participants, based on socio-demographics and relevant clinical variables are summarized in Table 1.

### Multiple Regression Analysis of the Acceptance of Multiple Regression Analysis With Acceptance as Dependent Variable

With a view to meeting this study's overriding goals of studying the relationships between predictor variables (anxiety toward pain, sensitivity to pain and to reinforcement, and the responses of significant persons to pain behaviors) on the dependent variable (pain acceptance), a multiple stepwise linear regression exploratory analysis has been conducted.

Table 2 shows the results of the correlations between the predictor variables and the acceptance of pain (CPAQ). The analyses revealed a model that added significant persons' punishing responses (ΔR^2^ = 0.045) when facing pain behaviors to other variables, such as pain-related anxiety (ΔR^2^ = 0.060) and reinforcement sensitivity (ΔR^2^ = 0.366). This led to a statistically significant model (*F* = 17.255, *p* ≤ 0.01) that explained 44% of the variance on the dependent variable (Adjusted R^2^ = 0.444). All the correlations that feature in the model were significant when predicting pain-related anxiety, and reached the statistical criterion *p* ≤ 0.05 required to do so. The linear regression analyses reveal a negative and significant correlation between the three predictors and the dependent variable, recording an effect size that varies from small to medium ranges according to Cohen's criteria (76).

**Table 2:** Multiple stepwise linear regression analysis of pain-related anxiety, sensitivity to punishment and reinforcement, and the punishing responses of significant persons toward pain behaviors, on the acceptance of pain*.

**Step**	**Predictors**						**Regression model**			
		***B***	**β**	***t***	***p***	**R**	**R^2^**	**ΔR^2^**	***F***	***P***
1	Constant PASS_20_total	96.226 −0.681	−0.605***	18.406 −5.891	0.00 0.00	0.36	0.356	0.366	34.698***	0.000
2	Constant Pass_20_total Reinforcement sensitivity	97.954 −0.548 −1.098	−0.488***−0.272*	19.346 −4.462 −2.489	0.00 0.00 0.016	0.42	0.407	0.060	21.948***	0.000
3	Constant Pass_20_total Reinforcement sensitivity Whympi punishment	99.688 −0.563 −1.086 −0.592	−0.501***−0.269*−0.212*	20.083 −4.722 −2.541 −2.222	0.00 0.00 0.14 0.030	0.47	0.444	0.045	17.255***	0.000

N = 62. *p < 0.05; ***p < 0.001; B = Non-standardized regression coefficient; Beta = Standardized regression coefficient; PASS_20_Total: Overall score of the pain anxiety symptoms scale; Reinforcement sensitivity: Reinforcement sensitivity subscale of the Sensitivity to Punishment and Sensitivity to Reward Questionnaire; Whympi punishment: Punishment responses to pain behavior in the second domain of the West Haven-Yale Multidimensional Pain Inventory.

## Discussion

The results presented here reveal that emotional variables such as pain-related anxiety, reinforcement sensitivity, and punishing responses toward pain behaviors by significant people for the patient accurately predict the individual's predisposition to accept pain. As noted, the scope of these relationship has generally been small or moderate. Regression analyses have provided us with a more profound understanding of the relationships between these variables described in the literature.

Pain-related anxiety has proven to be the best predictor of the acceptance of pain. The results are consistent with the findings reported by other scholars on a negative and robust correlation between pain-related anxiety and the components of its acceptance (11–13, 21, 35–37). This therefore highlights the importance that pain-related anxiety might have as a variable linked to the acquisition of fear and escape or avoidance behaviors in the face of pain, as reported by other scholars in the literature reviewed (39–41). According to pain-avoidance models (77, 78), escape behavior impedes an elaborative processing of the stimuli being avoided (e.g., sensorial aspects of pain, thoughts, emotions or sensations) (79), which leads to the acquisition of fear related to the pain itself, and a biased interpretation of the symptoms as threatening (25, 80). This means that if the patient is experiencing high levels of anxiety, it is reasonable to assume that this emotion is going to play an important role in the way the patient suffers and copes with the illness and, therefore, in their clinical treatment.

Our findings show that the STR variable is linked to a greater predisposition toward the acceptance of pain in the presence of higher levels of STR. The results are consistent with the findings reported in other studies, which have noted this variable's importance in participants with chronic pain (47, 53). Nevertheless, prior studies have indicated that participants with chronic pain are expected to have a greater level of activation in the BIS, and a lower one in the BAS, with a greater presence of avoidance behaviors (47–51). Knowing that the activation of both systems is related to SP and STR (81), we expected to find a direct and significant correlation between STR or an indirect correlation between PS and pain acceptance. Nevertheless, these results can be explained when we consider that the perception of reinforcement varies for each person and depends on their psychological state, their values and their goals (82). For example, it is logical that someone with a high STR and greater impulsiveness is more motivated to achieve goals and assign behavioral resources accordingly, although for such a person it might be harder to accept that the pain, or the incapacity associated with it, no longer permits them to do so. It therefore seems probable that this individual may cope by seeking immediate relief for their symptoms in order to resolve the interference in the short term; in other words, the individual will mobilize behavioral resources looking for negative reinforcement, and they are more than likely to record more escape or avoidance behaviors. It therefore seems reasonable to contend that the higher the STR and the greater the impulsiveness, the lower the predisposition to accept pain. Nonetheless, future researchers will be tasked with clarifying this variable's role regarding acceptance and coping in participants with chronic pain.

This study has also uncovered a negative and significant correlation between the punishing responses toward pain behaviors shown by the patient's carers and pain acceptance. These results coincide with other studies that predicted a worse adjustment to pain in the presence of adverse contingencies for the patient (58, 59, 62, 66). The results also coincide with the findings made by McCraken (68), who has reported that the punishing responses of significant people are negatively associated with the acceptance of pain. Therefore, as noted earlier, the social support of significant persons for pain behaviors seems to be a highly influential variable in acceptance processes in contexts of chronic pain. The paucity of studies on this matter calls for further research designed to extend the information on the relationship between these two variables.

The results forthcoming here prompt us to make a series of suggestions that could help to improve the care provided for these participants. Pain-related anxiety and reinforcement sensitivity are variables to be considered during the assessment process. Whenever high scores are observed in any of these variables, it would be advisable to use some technique (e.g., cognitive restructuring) to work on cognitive aspects or even consider the possibility of a more traditional intervention for correcting a mistaken interpretation of the symptoms, reduce the perception of threat, and boost active coping with the illness, as in Cognitive Behavioral Therapy (CBT), which has proven to be extremely effective in cases of chronic pain [e.g., (83–85)]. In the case of low scores for these variables, the initial choice of treatment could involve any intervention based on third-generation therapies, as both ACT and MBIs have proven to be effective in pain contexts (33, 86–88). The results obtained also refer to the importance of providing families with accurate information on the way patient's behave when dealing with pain and their relationship with the treatment, whereby they can support the patient in a non-interfering manner.

These results and the aforementioned conclusions should be considered within the context of some of their limitations. Firstly, the sample used here involved discarding several participants that did not meet the inclusion criteria, and the final cohort consisted solely of participants with rheumatoid arthritis. Future research should study the relationship between these variables and other groups of participants with chronic pain. Moreover, the final sample is small, particularly in the case of males, so other researchers are advised to employ broader samples in the future with a view to comparing results. It is also important to talk about methodological issues arising from the self-report measure used to assess the main carers' responses to their participants' pain behaviors. This instrument rates the carers' responses based on the individual's own subjective opinion. This perception may be influenced by other psychological variables, which means these data should be interpreted with some caution.

## Conclusions

In sum, variables such as pain-related anxiety, STR, and the punishing responses of significant people for the patient predict a lower acceptance of pain in participants with chronic pain. We may therefore infer the convenience of taking them into consideration during the assessment process in the first clinical contacts. In turn, prior knowledge of these variables may inform the decision-making on the intervention to be performed in each case, which could improve the efficacy or success of this care. Based on the results obtained, there is a need to investigate these variables in relation to the components of the acceptance of pain, given the part they play in the treatment to be followed with these participants.

## Data Availability Statement

The original contributions presented in the study are included in the article/Supplementary Material, further inquiries can be directed to the corresponding author.

## Ethics Statement

The studies involving human participants were reviewed and approved by CEIC Hospital Clínico San Carlos. The patients/participants provided their written informed consent to participate in this study.

## Author Contributions

LP contributed in the conception, design of the study, acquired the data, analyzed and interpreted the patient data, drafted the article and overseed the final version of the article before submission, and was a major contributor in writing the manuscript. MP-N had a relevant role in the conceptualization of the study, analysis and interpretation of the data, and drafting the article. MR contributed in the conception and design of the study. LR-R and LL contributed to acquisition of data. All authors read and approved the final manuscript.

## Conflict of Interest

The authors declare that the research was conducted in the absence of any commercial or financial relationships that could be construed as a potential conflict of interest.

## Abbreviations

ACT: Acceptance and Commitment Therapy
AMAPAR: Association of Participants with Rheumatoid Arthritis
AS: Anxiety Sensitivity
BAS: Behavioral Approach/Activation System
BIS: Behavioral Inhibition System
CBT: Cognitive Behavioral Therapy
CPAQ: Chronic Pain Acceptance Questionnaire
MBIs: Mindfulness-based interventions
PASS-20: Pain Anxiety Symptoms Scale
SP: Sensitivity to punishment
SPSRQ: The Sensitivity to Punishment and Sensitivity to Reward Questionnaire
STR: Sensitivity to reward
WHO: World Health Organization
WHYMPI: The West Haven-Yale Multidimensional Pain Inventory.
