# Supplementary material for: Anxiety, Reinforcement Sensitivity and Social Context in Accepting the Experience of Pain Among Rheumatoid Arthritis Patients
Source: Front Psychiatry. 2020 Nov 25;11:554990. doi: 10.3389/fpsyt.2020.554990 (PMC7723888; doi:10.3389/fpsyt.2020.554990)
Supplement: Supplementary file 1 [file Data_Sheet_1.doc]

REGRESSION
  /DESCRIPTIVES MEAN STDDEV CORR SIG N
  /MISSING LISTWISE
  /STATISTICS COEFF OUTS R ANOVA COLLIN TOL CHANGE ZPP
  /CRITERIA=PIN(.05) POUT(.10)
  /NOORIGIN
  /DEPENDENT CPAQ_Total
  /METHOD=STEPWISE SENSIB_SC SENSIB_SR PASS_Total PASS_Cognitivo PASS_Evitacion PASS_Miedo
    PASS_Ansiedad WHYMPI_Castigo WHYMPI_Solicitas WHYMPI_Distractoras Edad Inicio_enf Tr_actual
  /RESIDUALS DURBIN.


Regression


Notes	
Output Created	07-SEP-2020 16:38:15	
Comments		
Input	Data	/Users/l.pinel/Documents/Doctorado/Articulos nuevos/3/sent to magazine/Base rellenada-docto-todos-task1-2-solo-AR*.sav	
	Active Dataset	DataSet2	
	Filter	<none>	
	Weight	<none>	
	Split File	<none>	
	N of Rows in Working Data File	62	
Missing Value Handling	Definition of Missing	User-defined missing values are treated as missing.	
	Cases Used	Statistics are based on cases with no missing values for any variable used.	
Syntax	REGRESSION
  /DESCRIPTIVES MEAN STDDEV CORR SIG N
  /MISSING LISTWISE
  /STATISTICS COEFF OUTS R ANOVA COLLIN TOL CHANGE ZPP
  /CRITERIA=PIN(.05) POUT(.10)
  /NOORIGIN
  /DEPENDENT CPAQ_Total
  /METHOD=STEPWISE SENSIB_SC SENSIB_SR PASS_Total PASS_Cognitivo PASS_Evitacion PASS_Miedo
    PASS_Ansiedad WHYMPI_Castigo WHYMPI_Solicitas WHYMPI_Distractoras Edad Inicio_enf Tr_actual
  /RESIDUALS DURBIN.	
Resources	Processor Time	00:00:00,04	
	Elapsed Time	00:00:00,00	
	Memory Required	15184 bytes	
	Additional Memory Required for Residual Plots	0 bytes	


Descriptive Statistics	
	Mean	Std. Deviation	N	
CPAQ_TOTAL	66,65	14,261	62	
SENSIB_Sensibilidad_Castigo	11,26	5,248	62	
SENSIB_Sensibilidad_Refuerzo	6,81	3,534	62	
PASS_20_Total	43,47	12,685	62	
PASS_20_Cognitivo	13,23	4,568	62	
PASS_20_Evitacion	13,11	4,181	62	
PASS_20_Miedo	8,98	3,981	62	
PASS_20_Ansiedad	8,15	2,980	62	
WHYMPI_Respuestas_Castigo	2,02	5,117	62	
WHYMPI_Solicitas	20,90	13,370	62	
WHYMPI_Distractoras	9,10	6,939	62	
Edad del paciente (en años)	53,24	11,299	62	
Tiempo transcurrido desde que recibió el primer diagnóstico	4,23	1,247	62	
Tratamiento farmacológico actual	6,53	1,533	62	


Correlations	
	CPAQ_TOTAL	SENSIB_Sensibilidad_Castigo	SENSIB_Sensibilidad_Refuerzo	PASS_20_Total	PASS_20_Cognitivo	PASS_20_Evitacion	PASS_20_Miedo	PASS_20_Ansiedad	
Pearson Correlation	CPAQ_TOTAL	1,000	-,354	-,483	-,605	-,572	-,449	-,462	-,453	
	SENSIB_Sensibilidad_Castigo	-,354	1,000	,183	,361	,350	,244	,291	,269	
	SENSIB_Sensibilidad_Refuerzo	-,483	,183	1,000	,432	,419	,354	,260	,353	
	PASS_20_Total	-,605	,361	,432	1,000	,834	,787	,824	,774	
	PASS_20_Cognitivo	-,572	,350	,419	,834	1,000	,560	,554	,491	
	PASS_20_Evitacion	-,449	,244	,354	,787	,560	1,000	,468	,464	
	PASS_20_Miedo	-,462	,291	,260	,824	,554	,468	1,000	,665	
	PASS_20_Ansiedad	-,453	,269	,353	,774	,491	,464	,665	1,000	
	WHYMPI_Respuestas_Castigo	-,182	,166	-,012	-,054	,026	-,115	-,010	-,096	
	WHYMPI_Solicitas	-,031	,016	,079	,003	-,059	,145	-,046	-,037	
	WHYMPI_Distractoras	-,026	,090	,036	-,045	-,052	,010	-,128	,044	
	Edad del paciente (en años)	,110	-,174	-,102	-,139	-,154	,011	-,174	-,137	
	Tiempo transcurrido desde que recibió el primer diagnóstico	,068	-,169	-,094	-,255	-,167	-,011	-,362	-,327	
	Tratamiento farmacológico actual	,172	-,085	,056	-,160	-,237	-,150	,001	-,110	
Sig. (1-tailed)	CPAQ_TOTAL	.	,002	,000	,000	,000	,000	,000	,000	
	SENSIB_Sensibilidad_Castigo	,002	.	,077	,002	,003	,028	,011	,017	
	SENSIB_Sensibilidad_Refuerzo	,000	,077	.	,000	,000	,002	,021	,002	
	PASS_20_Total	,000	,002	,000	.	,000	,000	,000	,000	
	PASS_20_Cognitivo	,000	,003	,000	,000	.	,000	,000	,000	
	PASS_20_Evitacion	,000	,028	,002	,000	,000	.	,000	,000	
	PASS_20_Miedo	,000	,011	,021	,000	,000	,000	.	,000	
	PASS_20_Ansiedad	,000	,017	,002	,000	,000	,000	,000	.	
	WHYMPI_Respuestas_Castigo	,078	,099	,464	,339	,419	,187	,470	,229	
	WHYMPI_Solicitas	,407	,452	,271	,489	,324	,130	,361	,387	
	WHYMPI_Distractoras	,420	,244	,392	,364	,344	,468	,161	,366	
	Edad del paciente (en años)	,198	,088	,214	,141	,115	,466	,088	,144	
	Tiempo transcurrido desde que recibió el primer diagnóstico	,299	,094	,233	,023	,097	,465	,002	,005	
	Tratamiento farmacológico actual	,090	,257	,334	,106	,032	,122	,496	,196	
N	CPAQ_TOTAL	62	62	62	62	62	62	62	62	
	SENSIB_Sensibilidad_Castigo	62	62	62	62	62	62	62	62	
	SENSIB_Sensibilidad_Refuerzo	62	62	62	62	62	62	62	62	
	PASS_20_Total	62	62	62	62	62	62	62	62	
	PASS_20_Cognitivo	62	62	62	62	62	62	62	62	
	PASS_20_Evitacion	62	62	62	62	62	62	62	62	
	PASS_20_Miedo	62	62	62	62	62	62	62	62	
	PASS_20_Ansiedad	62	62	62	62	62	62	62	62	
	WHYMPI_Respuestas_Castigo	62	62	62	62	62	62	62	62	
	WHYMPI_Solicitas	62	62	62	62	62	62	62	62	
	WHYMPI_Distractoras	62	62	62	62	62	62	62	62	
	Edad del paciente (en años)	62	62	62	62	62	62	62	62	
	Tiempo transcurrido desde que recibió el primer diagnóstico	62	62	62	62	62	62	62	62	
	Tratamiento farmacológico actual	62	62	62	62	62	62	62	62	

Correlations	
	WHYMPI_Respuestas_Castigo	WHYMPI_Solicitas	WHYMPI_Distractoras	Edad del paciente (en años)	Tiempo transcurrido desde que recibió el primer diagnóstico	Tratamiento farmacológico actual	
Pearson Correlation	CPAQ_TOTAL	-,182	-,031	-,026	,110	,068	,172	
	SENSIB_Sensibilidad_Castigo	,166	,016	,090	-,174	-,169	-,085	
	SENSIB_Sensibilidad_Refuerzo	-,012	,079	,036	-,102	-,094	,056	
	PASS_20_Total	-,054	,003	-,045	-,139	-,255	-,160	
	PASS_20_Cognitivo	,026	-,059	-,052	-,154	-,167	-,237	
	PASS_20_Evitacion	-,115	,145	,010	,011	-,011	-,150	
	PASS_20_Miedo	-,010	-,046	-,128	-,174	-,362	,001	
	PASS_20_Ansiedad	-,096	-,037	,044	-,137	-,327	-,110	
	WHYMPI_Respuestas_Castigo	1,000	-,298	-,231	,063	-,109	,116	
	WHYMPI_Solicitas	-,298	1,000	,758	,009	,224	,122	
	WHYMPI_Distractoras	-,231	,758	1,000	-,096	,141	,108	
	Edad del paciente (en años)	,063	,009	-,096	1,000	,273	-,125	
	Tiempo transcurrido desde que recibió el primer diagnóstico	-,109	,224	,141	,273	1,000	-,158	
	Tratamiento farmacológico actual	,116	,122	,108	-,125	-,158	1,000	
Sig. (1-tailed)	CPAQ_TOTAL	,078	,407	,420	,198	,299	,090	
	SENSIB_Sensibilidad_Castigo	,099	,452	,244	,088	,094	,257	
	SENSIB_Sensibilidad_Refuerzo	,464	,271	,392	,214	,233	,334	
	PASS_20_Total	,339	,489	,364	,141	,023	,106	
	PASS_20_Cognitivo	,419	,324	,344	,115	,097	,032	
	PASS_20_Evitacion	,187	,130	,468	,466	,465	,122	
	PASS_20_Miedo	,470	,361	,161	,088	,002	,496	
	PASS_20_Ansiedad	,229	,387	,366	,144	,005	,196	
	WHYMPI_Respuestas_Castigo	.	,009	,035	,314	,201	,185	
	WHYMPI_Solicitas	,009	.	,000	,471	,040	,173	
	WHYMPI_Distractoras	,035	,000	.	,230	,136	,203	
	Edad del paciente (en años)	,314	,471	,230	.	,016	,167	
	Tiempo transcurrido desde que recibió el primer diagnóstico	,201	,040	,136	,016	.	,110	
	Tratamiento farmacológico actual	,185	,173	,203	,167	,110	.	
N	CPAQ_TOTAL	62	62	62	62	62	62	
	SENSIB_Sensibilidad_Castigo	62	62	62	62	62	62	
	SENSIB_Sensibilidad_Refuerzo	62	62	62	62	62	62	
	PASS_20_Total	62	62	62	62	62	62	
	PASS_20_Cognitivo	62	62	62	62	62	62	
	PASS_20_Evitacion	62	62	62	62	62	62	
	PASS_20_Miedo	62	62	62	62	62	62	
	PASS_20_Ansiedad	62	62	62	62	62	62	
	WHYMPI_Respuestas_Castigo	62	62	62	62	62	62	
	WHYMPI_Solicitas	62	62	62	62	62	62	
	WHYMPI_Distractoras	62	62	62	62	62	62	
	Edad del paciente (en años)	62	62	62	62	62	62	
	Tiempo transcurrido desde que recibió el primer diagnóstico	62	62	62	62	62	62	
	Tratamiento farmacológico actual	62	62	62	62	62	62	


Variables Entered/Removeda	
Model	Variables Entered	Variables Removed	Method	
1	PASS_20_Total	.	Stepwise (Criteria: Probability-of-F-to-enter <= ,050, Probability-of-F-to-remove >= ,100).	
2	SENSIB_Sensibilidad_Refuerzo	.	Stepwise (Criteria: Probability-of-F-to-enter <= ,050, Probability-of-F-to-remove >= ,100).	
3	WHYMPI_Respuestas_Castigo	.	Stepwise (Criteria: Probability-of-F-to-enter <= ,050, Probability-of-F-to-remove >= ,100).	

a. Dependent Variable: CPAQ_TOTAL	


Model Summaryd	
Model	R	R Square	Adjusted R Square	Std. Error of the Estimate	Change Statistics	
					R Square Change	F Change	df1	df2	
1	,605a	,366	,356	11,446	,366	34,698	1	60	
2	,653b	,427	,407	10,980	,060	6,194	1	59	
3	,687c	,472	,444	10,631	,045	4,939	1	58	

Model Summaryd	
Model	Change Statistics	Durbin-Watson	
	Sig. F Change		
1	,000		
2	,016		
3	,030	1,656	

a. Predictors: (Constant), PASS_20_Total	
b. Predictors: (Constant), PASS_20_Total, SENSIB_Sensibilidad_Refuerzo	
c. Predictors: (Constant), PASS_20_Total, SENSIB_Sensibilidad_Refuerzo, WHYMPI_Respuestas_Castigo	
d. Dependent Variable: CPAQ_TOTAL	


ANOVAa	
Model	Sum of Squares	df	Mean Square	F	Sig.	
1	Regression	4545,751	1	4545,751	34,698	,000b	
	Residual	7860,443	60	131,007			
	Total	12406,194	61				
2	Regression	5292,535	2	2646,268	21,948	,000c	
	Residual	7113,658	59	120,570			
	Total	12406,194	61				
3	Regression	5850,801	3	1950,267	17,255	,000d	
	Residual	6555,392	58	113,024			
	Total	12406,194	61				

a. Dependent Variable: CPAQ_TOTAL	
b. Predictors: (Constant), PASS_20_Total	
c. Predictors: (Constant), PASS_20_Total, SENSIB_Sensibilidad_Refuerzo	
d. Predictors: (Constant), PASS_20_Total, SENSIB_Sensibilidad_Refuerzo, WHYMPI_Respuestas_Castigo	


Coefficientsa	
Model	Unstandardized Coefficients	Standardized Coefficients	t	Sig.	Correlations	
	B	Std. Error	Beta			Zero-order	
1	(Constant)	96,226	5,228		18,406	,000		
	PASS_20_Total	-,681	,116	-,605	-5,891	,000	-,605	
2	(Constant)	97,954	5,063		19,346	,000		
	PASS_20_Total	-,548	,123	-,488	-4,462	,000	-,605	
	SENSIB_Sensibilidad_Refuerzo	-1,098	,441	-,272	-2,489	,016	-,483	
3	(Constant)	99,688	4,964		20,083	,000		
	PASS_20_Total	-,563	,119	-,501	-4,722	,000	-,605	
	SENSIB_Sensibilidad_Refuerzo	-1,086	,427	-,269	-2,541	,014	-,483	
	WHYMPI_Respuestas_Castigo	-,592	,266	-,212	-2,222	,030	-,182	

Coefficientsa	
Model	Correlations	
	Partial	Part	Tolerance	VIF	
1	(Constant)					
	PASS_20_Total	-,605	-,605	1,000	1,000	
2	(Constant)					
	PASS_20_Total	-,502	-,440	,813	1,230	
	SENSIB_Sensibilidad_Refuerzo	-,308	-,245	,813	1,230	
3	(Constant)					
	PASS_20_Total	-,527	-,451	,811	1,233	
	SENSIB_Sensibilidad_Refuerzo	-,317	-,243	,813	1,230	
	WHYMPI_Respuestas_Castigo	-,280	-,212	,997	1,003	

a. Dependent Variable: CPAQ_TOTAL	


Excluded Variablesa	
Model	Beta In	t	Sig.	Partial Correlation	Collinearity Statistics	
					Tolerance	VIF	Minimum Tolerance	
1	SENSIB_Sensibilidad_Castigo	-,156b	-1,426	,159	-,183	,869	1,150	,869	
	SENSIB_Sensibilidad_Refuerzo	-,272b	-2,489	,016	-,308	,813	1,230	,813	
	PASS_20_Cognitivo	-,221b	-1,190	,239	-,153	,305	3,283	,305	
	PASS_20_Evitacion	,072b	,429	,669	,056	,380	2,630	,380	
	PASS_20_Miedo	,114b	,628	,532	,082	,322	3,109	,322	
	PASS_20_Ansiedad	,039b	,236	,814	,031	,402	2,490	,402	
	WHYMPI_Respuestas_Castigo	-,216b	-2,158	,035	-,270	,997	1,003	,997	
	WHYMPI_Solicitas	-,028b	-,275	,785	-,036	1,000	1,000	1,000	
	WHYMPI_Distractoras	-,054b	-,519	,606	-,067	,998	1,002	,998	
	Edad del paciente (en años)	,026b	,252	,802	,033	,981	1,020	,981	
	Tiempo transcurrido desde que recibió el primer diagnóstico	-,092b	-,862	,392	-,112	,935	1,069	,935	
	Tratamiento farmacológico actual	,077b	,737	,464	,096	,974	1,026	,974	
2	SENSIB_Sensibilidad_Castigo	-,148c	-1,407	,165	-,182	,868	1,151	,731	
	PASS_20_Cognitivo	-,171c	-,948	,347	-,123	,300	3,329	,296	
	PASS_20_Evitacion	,082c	,511	,611	,067	,380	2,632	,353	
	PASS_20_Miedo	,034c	,191	,849	,025	,310	3,224	,271	
	PASS_20_Ansiedad	,051c	,327	,745	,043	,401	2,493	,373	
	WHYMPI_Respuestas_Castigo	-,212c	-2,222	,030	-,280	,997	1,003	,811	
	WHYMPI_Solicitas	-,007c	-,074	,941	-,010	,993	1,007	,807	
	WHYMPI_Distractoras	-,039c	-,390	,698	-,051	,994	1,006	,810	
	Edad del paciente (en años)	,015c	,146	,885	,019	,979	1,022	,804	
	Tiempo transcurrido desde que recibió el primer diagnóstico	-,087c	-,853	,397	-,111	,935	1,070	,767	
	Tratamiento farmacológico actual	,114c	1,135	,261	,147	,955	1,047	,779	
3	SENSIB_Sensibilidad_Castigo	-,107d	-1,020	,312	-,134	,834	1,199	,721	
	PASS_20_Cognitivo	-,123d	-,696	,490	-,092	,295	3,385	,291	
	PASS_20_Evitacion	,042d	,268	,790	,035	,375	2,669	,352	
	PASS_20_Miedo	,059d	,342	,734	,045	,309	3,237	,269	
	PASS_20_Ansiedad	,023d	,148	,883	,020	,398	2,512	,372	
	WHYMPI_Solicitas	-,078d	-,778	,440	-,103	,903	1,107	,806	
	WHYMPI_Distractoras	-,094d	-,954	,344	-,125	,939	1,065	,805	
	Edad del paciente (en años)	,027d	,276	,784	,037	,975	1,025	,802	
	Tiempo transcurrido desde que recibió el primer diagnóstico	-,117d	-1,179	,243	-,154	,920	1,087	,762	
	Tratamiento farmacológico actual	,139d	1,430	,158	,186	,944	1,059	,778	

a. Dependent Variable: CPAQ_TOTAL	
b. Predictors in the Model: (Constant), PASS_20_Total	
c. Predictors in the Model: (Constant), PASS_20_Total, SENSIB_Sensibilidad_Refuerzo	
d. Predictors in the Model: (Constant), PASS_20_Total, SENSIB_Sensibilidad_Refuerzo, WHYMPI_Respuestas_Castigo	


Collinearity Diagnosticsa	
Model	Dimension	Eigenvalue	Condition Index	Variance Proportions	
				(Constant)	PASS_20_Total	SENSIB_Sensibilidad_Refuerzo	WHYMPI_Respuestas_Castigo	
1	1	1,961	1,000	,02	,02			
	2	,039	7,051	,98	,98			
2	1	2,839	1,000	,01	,01	,02		
	2	,123	4,809	,15	,05	,92		
	3	,038	8,668	,84	,95	,06		
3	1	3,016	1,000	,01	,01	,02	,02	
	2	,824	1,913	,00	,00	,01	,96	
	3	,122	4,969	,15	,05	,92	,01	
	4	,037	8,985	,84	,94	,06	,01	

a. Dependent Variable: CPAQ_TOTAL	


Residuals Statisticsa	
	Minimum	Maximum	Mean	Std. Deviation	N	
Predicted Value	36,70	82,40	66,65	9,794	62	
Residual	-26,118	26,979	,000	10,367	62	
Std. Predicted Value	-3,058	1,609	,000	1,000	62	
Std. Residual	-2,457	2,538	,000	,975	62	

a. Dependent Variable: CPAQ_TOTAL	
